# Single‐Step Biofabrication of In Situ Spheroid‐Forming Compartmentalized Hydrogel for Clinical‐Sized Cartilage Tissue Formation

**DOI:** 10.1002/adhm.202300095

**Published:** 2023-10-26

**Authors:** Bas van Loo, Maik Schot, Melvin Gurian, Tom Kamperman, Jeroen Leijten

**Affiliations:** ^1^ Department of Developmental BioEngineering Faculty of Science and Technology Technical Medical Centre University of Twente Drienerlolaan 5 Enschede 7522 NB The Netherlands; ^2^ IamFluidics B.V. De Veldmaat 17 Enschede 7522 NM The Netherlands

**Keywords:** aggregate, biofabrication, cell encapsulation, compartmentalized hydrogels, in‐air microfluidics, microfluidics, spheroids

## Abstract

3D cellular spheroids offer more biomimetic microenvironments than conventional 2D cell culture technologies, which has proven value for many tissue engineering applications. Despite beneficiary effects of 3D cell culture, clinical translation of spheroid tissue engineering is challenged by limited scalability of current spheroid formation methods. Although recent adoption of droplet microfluidics can provide a continuous production process, use of oils and surfactants, generally low throughput, and requirement of additional biofabrication steps hinder clinical translation of spheroid culture. Here, the use of clean (e.g., oil‐free and surfactant‐free), ultra‐high throughput (e.g., 8.5 mL min^−1^, 10 000 spheroids s^−1^), single‐step, in‐air microfluidic biofabrication of spheroid forming compartmentalized hydrogels is reported. This novel technique can reliably produce 1D fibers, 2D planes, and 3D volumes compartmentalized hydrogel constructs, which each allows for distinct (an)isotropic orientation of hollow spheroid‐forming compartments. Spheroids produced within ink‐jet bioprinted compartmentalized hydrogels outperform 2D cell cultures in terms of chondrogenic behavior. Moreover, the cellular spheroids can be harvested from compartmentalized hydrogels and used to build shape‐stable centimeter‐sized biomaterial‐free living tissues in a bottom‐up manner. Consequently, it is anticipated that in‐air microfluidic production of spheroid‐forming compartmentalized hydrogels can advance production and use of cellular spheroids for various biomedical applications.

## Graphical abstract

In air microfluidics allows for successful production of cell‐laden hollow compartmentalized hydrogels and enables in situ formation of cellular spheroids in an ultra‐high throughput, clean, and single‐step manner. Utilizing the ultra‐high throughput of in air microfluidics, scalable production of spheroid‐based clinically sized cartilage tissues is realized in relevant timeframes, which represents a significant step toward clinical translation of cellular spheroids.

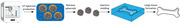

## 1 Introduction

2D in vitro cell culture has historically been the gold standard in the field of tissue engineering, which aims to repair, regenerate, or replace damaged living tissues. However, conventional 2D cell culture environments do not resemble their natural in vivo counterpart and thus adversely affect cellular behavior.^[^ ^1^, ^2^, ^3^, ^4^, ^5^ ^]^ 3D cell cultures such as cellular spheroid or organoids have allowed for a more biomimetic approach toward cell culture.^[^ ^6^, ^7^, ^8^ ^]^ It has been widely reported that biomimetic 3D spheroid cultured cells outperform 2D cultured cells when utilized for tissue engineering,^[^ ^9^, ^10^, ^11^, ^12^, ^13^ ^]^ and can facilitate more reliable drug target discovery and enable engineering of macroscopic tissue constructs with improved functionalities.^[^ ^14^, ^15^, ^16^, ^17^, ^18^, ^19^ ^]^ For example, 3D printing of stem cell spheroid‐laden bioinks improves functional behavior (e.g., chondrogenic differentiation and cartilage matrix deposition) of biofabricated constructs.^[^ ^20^ ^]^ Although promising, 3D culture techniques such as spheroid culture are hindered in their clinical translation by their production methods, which are often batch processes that are characterized by low production rates. These batch production technologies such as microwells^[^ ^9^, ^10^, ^12^, ^21^, ^22^, ^23^, ^24^ ^]^ and hanging drops^[^ ^13^, ^25^, ^26^, ^27^ ^]^ require multiple complex and time‐consuming steps that only offer a limited yield of spheroids. Although specific bioreactor approaches could allow for higher production yields, they are still limited to being batch processes that often associate with low levels of control of spheroid diameter as compared to compartmentalized technologies such as microwells and hanging drops.^[^ ^28^ ^]^ While this might be sufficient for the small lab‐scale experimentation, a robust single‐step biofabrication technology with high production rates and high monodispersity remains needed to facilitate the clinical translation of spheroid technology for clinically sized tissues.^[^ ^29^, ^30^ ^]^

Microfluidic compartmentalization has recently been explored to evolve spheroid production from a batch process toward a continuous process. Microfluidic encapsulation of cells in hollow biomaterial compartments allows for the controlled continuous production of cellular spheroid‐forming microbioreactors, which offers a higher production rate than conventional batch‐based processes.^[^ ^31^, ^32^, ^33^, ^34^, ^35^, ^36^, ^37^, ^38^, ^39^ ^]^ However, the design of conventional on‐chip microfluidic droplet generators prevents the widespread clinical use of microfluidic produced spheroids owing to a variety of reasons. First, conventional on‐chip microfluidics requires the use of immiscible fluids for droplet formation which often necessitates the use of oils and surfactants that are known to be potentially harmful and often incompatible with clinical applications.^[^ ^40^, ^41^, ^42^, ^43^, ^44^ ^]^ Produced spheroid‐forming compartments therefore need extensive washing in an attempt to remove the oil and surfactants before culture/use, which associates with time‐demanding artisanal processes that adversely affect cell viability.^[^ ^45^, ^46^ ^]^ Second, while conventional on‐chip microfluidics allows for continuous spheroid production, droplet formation is often limited to the dripping regime resulting in low throughputs (<10 µL min^−1^), which is still insufficient for most clinical applications.^[^ ^47^ ^]^ Lastly, retrieving spheroids from their compartments to enable further biofabrication processing typically requires a complex multistep process that can adversely affect cell survival.^[^ ^44^ ^]^ Consequently, a clean, fast, cell‐friendly, and single‐step biofabrication strategy that can endow large engineered tissues with in situ spheroid‐forming properties has remained wanted.

Here, we introduce a novel in air microfluidics (IAMF)^[^ ^48^ ^]^ based bioprinting technique that overcomes the translational limitations of spheroid use. Specifically, IAMF enabled the engineering of living matter that contained biomaterial‐free cell‐laden compartments, which acted as spheroid‐forming microreactors. Advantageously, this innovative approach represents a single‐step biofabrication technique for the engineering of clinically sized hydrogels that contain a high density of cellular spheroids that are produced at clinically relevant rates (1–8.5 mL min^−1^ equaling 10 000 spheroids s^−1^). This significant increase in production throughput is achieved by IAMF's ability to monodispersly endow ink‐jet bioprinted bioinks with hollow compartments in the jetting regime, while conventional on‐chip approaches are limited to the much slower dripping regime.^[^ ^49^ ^]^ This novel biofabrication process is also remarkably clean as IAMF obviates the traditional need for oils, surfactants, or sacrificial templates to create the hollow compartments within engineered tissues. In addition to conventional machine‐based ink‐jet bioprinting, we demonstrated that our in‐line bottom‐up biofabrication could also be used in the form of a simple handheld device to manually print spheroid‐forming compartmentalized hydrogels with multiple complexities, which further facilitates the technology's compatibility with clinical applications. While the translational challenges of upscaled production of microtissues are important for a plethora of distinct tissue types,^[^ ^28^ ^]^ we produced clinical‐sized cartilage tissues to function as a model tissue while acknowledging that a wide range of other (organ) shapes could also be produced using this approach.

## 2 Results and Discussion

### 2.1 In Air Microfluidics for Compartmentalized Hydrogel Biofabrication

**Figure 1:**
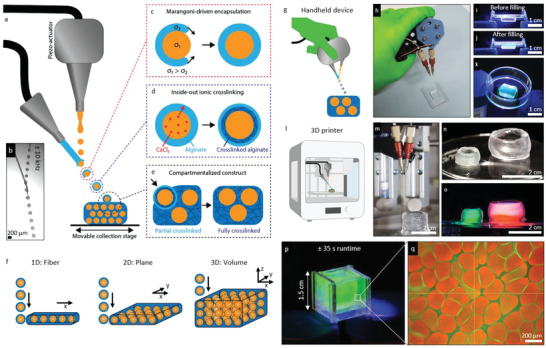
In air microfluidic biofabrication of highly compartmentalized hydrogels. a) Schematic of compartmentalized hydrogel production process using a two nozzle in air microfluidics approach. b) Micrograph of droplet formation and droplet/compartment encapsulation in air. c) Surface tension Marangoni flow enables encapsulation of the core droplet by a hydrogel precursor solution. d) CaCl_2_ diffuses from the inside of the two‐layered all‐aqueous droplet toward the alginate layer, allowing for an inside‐out ionic crosslinking mechanism, which results in crosslinked alginate at the interface between the two droplet layers, while the crosslinking in the shell remains ongoing. e) The ongoing crosslinking in the microcompartment's shell upon impact was leveraged to engineer solid multicompartmentalized hydrogels. f) Controlled deposition of compartments allowed for 1D fiber, 2D planes, and 3D volumes of compartmentalized hydrogels. g) Schematic and h) photograph of a 3D printer with integrated in air microfluidic setup. i) Photograph of hollow tube‐like compartmentalized hydrogel structures printed using a 3D printer that j) are nonleaking as visualized by containment of dextran‐FITC (green) and dextran‐TRITC (red) staining solutions. k) Schematic of handheld IAMF device for manual ink‐jet bioprinting of compartmentalized hydrogel bioinks. l) Photograph of handheld IAMF device being used for direct defect filling of a plastic mold. Fluorescent photographs of the mold m) before and n) after being conformally filled with compartmentalized hydrogel via handheld ink‐jet bioprinting, o) which was demonstrated shape stable even after being removed from the mold. p) Photograph of compartmentalized fluorescent hydrogel produced with a production rate of 8.5 mL min^−1^, which enabled the filling of a 1.5 × 1.5 × 1.5 cm mold in 35 s. q) Confocal fluorescence microscopy confirmed the formation of compartmentalized hydrogels, with alginate stained in green with dextran‐FITC and hollow core stained in red with dextran‐TRITC.

IAMF is a microfluidic approach that enables controlled micrometer‐sized droplet production by in‐air colliding a liquid jet with a constant period stream of aqueous droplets that were created by piezo‐actuating a liquid jet, which allows for chip‐free, oil‐free, and cytocompatible production of monodisperse microparticles at ultra‐high throughputs.^[^ ^48^ ^]^ In this study, we combined IAMF with ink‐jet bioprinting to allow for controlled merging of in‐air formed hollow microcapsules that enable the production of large‐scale compartmentalized hydrogels. To this end, a two micronozzle setup was used in combination with a moveable *XYZ* collection stage (**Figure** **1**a).

To form hollow microcapsules, a core microjet solution containing calcium chloride (CaCl_2_) was collided with a controlled droplet train that was produced by superimposing a vibration onto the micronozzle using piezo‐actuation (Figure 1b). Drop‐jet coalescence with an alginate precursor microjet with lowered surface tension (*σ*_core_ > *σ*_alginate_) allows for Marangoni‐driven encapsulation (Figure 1c). This encapsulation process occurs on a time scale of *τ*_e_ ∼ (*ρµD* ^4^/Δ*σ* ^2^)^1/3^, with *ρ*, *D*, *µ*, and *σ* denoting the microjet density, diameter, viscosity, and surface tension, respectively, which is typically within a few milliseconds.^[^ ^48^ ^]^ This allows for complete encapsulation within the 10 to 100 ms droplet flight time before the droplets are collected. This enables the production of two‐layered droplets containing a CaCl_2_ core and an alginate precursor shell. The resulting core–shell (i.e., calcium‐alginate) compound droplets were crosslinked in an inside‐out manner by CaCl_2_ diffusion from the core layer toward the alginate layer (Figure 1d). Since ionic alginate crosslinking occurs in the range of milliseconds^[^ ^50^ ^]^ and because of the inside‐out nature of the crosslinking, the inner alginate layer was crosslinked while the compound droplet was still in the air, while the outer alginate layer crosslinking occurred over time via inside‐out diffusion of Ca^2+^ ions. The partially crosslinked microcapsules were then collected in a controlled manner by either a moveable *XYZ* collection or micronozzle stage. Of note, the moment of impact was timed such that the crosslinking of the in‐air‐formed microcapsules was still ongoing allowing them to crosslink with the microcapsules came in physical contact with immediately upon landing thereby effectively forming an instantaneously solid 3D construct containing hollow microcompartments (Figure 1e).

We hypothesized that 1D fibers, 2D planes, and 3D volumes of compartmentalized hydrogels could be formed in a robust, predictable, and controllable manner by tuning microcapsule placement using standard droplet‐based biofabrication methods (Figure 1f). This biofabrication approach was demonstrated using a handheld bioprinting device (Figure 1g,h), which allowed for manual direct defect‐filling using the shape‐stable compartmentalized hydrogel (Figure 1i **–**k). The handheld approach is anticipated to facilitate clinical applications such as in situ bioprinting during surgical procedures. Moreover, we demonstrated that this novel biofabrication approach could be also combined with programmable 3D bioprinters (Figure 1l,m), which we demonstrated allowed for the production of large‐scale structures composed of more complex geometries such as shape‐stable tube‐like compartmentalized hydrogel (Figure 1n,o and Movie S1, Supporting Information). The ultra‐high throughput nature of IAMF (up to 8.5 mL min^−1^) (Figure S1, Supporting Information) allows for the biofabrication of clinically sized compartmentalized hydrogels in a fast, direct, and single‐step manner (Figure 1p,q).

Owing to the ultra‐high throughputs and short in‐air flight time of IAMF produced droplets, a fast (millisecond) crosslinking strategy such as the proposed ionic crosslinking of alginate or photopolymerization is typically required.^[^ ^51^ ^]^ However, materials relying on slower (seconds to minutes) crosslinking mechanisms such as silk fibroin can still be utilized by leveraging alginate as sacrificial structural interpenetrating network template.^[^ ^52^ ^]^

### 2.2 Size, Shape, and Isotropic Control of Microcompartments and Macrogel using In Air Microfluidic Biofabrication

**Figure 2:**
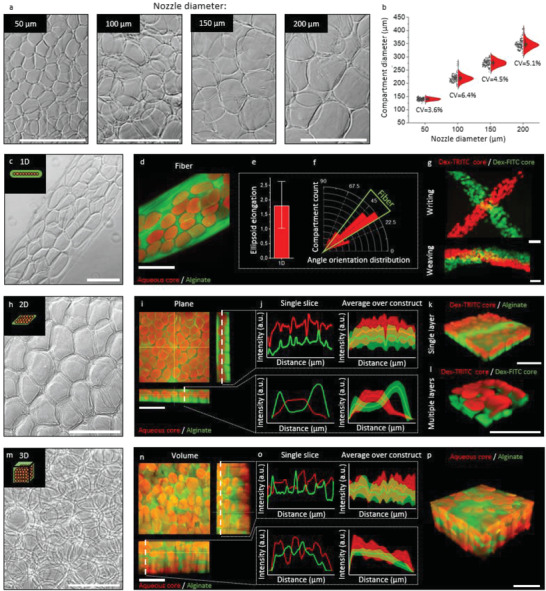
Controlled yet distinct morphologies in 1D, 2D, and 3D compartmentalized hydrogels produced by in air microfluidic biofabrication. a) Micrographs of hollow alginate microcapsules that were produced using a variety of nozzle with distinct diameters, which effectively controlled the hydrogel compartment's diameter b) with high monodispersity (*n* = 150). c) Brightfield micrograph of 1D compartmentalized fiber. d) Fluorescent confocal micrograph of compartmentalized fiber with alginate stained green with dextran‐FITC and compartments red with dextran‐TRITC. e) Quantification of compartment alignment as compared to its compartmentalized fiber (*n* = 54). f) Ellipsoid elongation of compartments in 1D fibers as compared to the 3D volume of its compartmentalized hydrogel (*n* = 54). g) Fluorescent confocal micrograph of compartmentalized fibers used for writing and weaving with multiple compartment payloads (green dextran‐FITC and red dextran‐TRITC). h) Brightfield micrograph of a 2D compartmentalized hydrogel sheet. i) Fluorescent confocal *z*‐stack of compartmentalized sheet with alginate stained green using dextran‐FITC and compartments stained red using dextran‐TRITC. j) Fluorescence intensity histograms of compartmentalized plane along the *xy*‐plane (top) and *x*‐plane (bottom) for a single slice (left) and as an average over the construct (right). k) Fluorescent confocal 3D reconstruction of a single layer compartmentalized sheet with alginate stained green using dextran‐FITC and compartments stained red using dextran‐TRITC. l) Fluorescent confocal 3D reconstruction of a double layer compartmentalized plane with multiple compartment payloads (green dextran‐FITC and red dextran‐TRITC). m) Brightfield micrograph of a 3D compartmentalized hydrogel volume. n) Fluorescent confocal *z*‐stack of compartmentalized volume with alginate stained green using dextran‐FITC and compartments stained red using dextran‐TRITC. o) Fluorescence intensity histograms of compartmentalized volume along the *xy*‐plane (top) and *x*‐plane (bottom) for a single slice (left) and as an average over the construct (right). p) Fluorescent confocal 3D reconstruction of a single layer of compartmentalized hydrogel volume with alginate stained green using dextran‐FITC and compartments stained red using dextran‐TRITC. Scale bars equal 500 µm. Data are presented as mean ± SD.

The size and shape of alginate hydrogel as well as their incorporated hollow compartments were controlled based on the placement of different‐sized micronozzles on a moveable *XYZ* stage. Micronozzles with an inner diameter of 50, 100, 150, and 200 µm produced monodisperse (CV < 10%) compartment sizes of 140 ± 5, 219 ± 14, 277 ± 12, and 347 ± 18 µm, respectively (**Figure** **2**a,b). This represents a significant improvement over conventional high throughput technologies such as large bioreactors which are often associated with polydispersity of tissue sizes.^[^ ^28^ ^]^

Compartmentalized hydrogels with distinct dimensions such as 1D fibers, 2D sheets, and 3D volumes can be achieved through movement of micronozzles in multiple dimensions with theoretical resolutions similar to the compartment sizes (140 ± 5, 219 ± 14, 277 ± 12, and 347 ± 18 µm for core jet micronozzles with an inner diameter of 50, 100, 150, and 200 µm, respectively). The ability to produce these fundamental shapes offers the opportunity for fabrication of larger arbitrary‐shaped tissues by further assembling these shapes.^[^ ^53^ ^]^ Compartmentalized fibers were produced by movement of the micronozzles in a single dimension (Figure 2c,d). Uniquely, this created hydrogel fibers containing ellipsoidal compartments with an elongation ratio of 1.7 +/− 0.5 (Figure 2e), which were consistently oriented with their ellipsoidal shape following the fiber's orientation (Figure 2f). This compartment shape control offers interesting possibilities since it is known that endowing elongation ratios in physical space in which microtissues are cultured/grown result in consistent and distinct behaviors that guide among others morphogenesis.^[^ ^54^ ^]^ Furthermore, multiple material writings can be formed with this biofabrication technique using a variety of payloads in microcompartments by utilizing the moveable micronozzle stage in 2D as demonstrated using dextran‐TRITC (tetramethylrhodamine isothiocyanate) and dextran‐FITC (fluorescein isothiocyanate, Figure 2g). Moreover, formed fibers were associated with good handleability (e.g., facile manual manipulation), which could potentially be used for applications such as weaving (Figure 2g), knitting, and braiding without inevitable fiber failure.^[^ ^55^, ^56^, ^57^, ^58^ ^]^ The ability to align cell‐laden fibers has been reported to be capable of improving tissue engineering applications in which alignment is important such as muscle tissue engineering applications.^[^ ^59^, ^60^, ^61^ ^]^ Additionally, cell‐laden microfibers are also utilized for islet cell encapsulation, making use of the easy retrievability of fibers when implanted in a patient body.^[^ ^55^, ^62^ ^]^

While moving the micronozzles in a single dimension creates hydrogel microfibers, a 2D movement allowed for the formation of spatially organized single‐layer hydrogel sheets (Figure 2h). Confocal *z*‐stack analysis confirmed the hollow (e.g., aqueous and noncrosslinked) nature of the compartments within the printed hydrogel sheet (Figure 2i). Notably, the formed hydrogel sheets were characterized by a symmetrical yet anisotropic nature, which was corroborated by semiquantitative histograms analysis of individual confocal slices (Figure 2j). Averaging histogram data of the *X* or *Y* plane indicated random placement of compartments, while the *Z* plane was shown to be consistently organized in terms of compartment placement at a specific height. Complexity in the planes such as multilayers and multiple materials can be added by repeating the controlled process of plane formation, which results in hydrogels composed of multiple layered planes, which provides the potential to use multiple materials or material properties in a height defined manner (Figure 2k,l). 2D cell‐laden sheets can function as patches, which have been proven to be successful for, among others, cardiac tissue engineering applications and wound healing patches^[^ ^63^, ^64^, ^65^ ^]^ and can be rolled up to mimic tubular structures.^[^ ^66^ ^]^

In‐air printing of annealing microcapsules was also demonstrated to be readily capable of rapidly creating voluminous 3D compartmentalized hydrogels (Figure 2m,n). 3D hydrogels containing spheroids have been shown to be relevant for a wide variety of biomedical applications, e.g., it has consistently been shown to increase chondrogenesis and cartilage formation.^[^ ^10^, ^12^ ^]^ While compartment placing in 2D compartmentalized sheets was reproducibly anisotropic, the average over construct histograms of 3D volumes revealed that 3D volumes were characterized by isotropic compartment placement (Figure 2o,p). Packing density (compartments/volume) can be controlled by tuning the compartment sizes through the use of core jet micronozzles with different inner diameters as well as via the relative flow rates. No kinetic displacement of the fabricated structure upon collision with newly jetted compartments has been observed.

In‐air microfluidics biofabrication of compartmentalized hydrogels of 1D, 2D, and 3D materials offers, among others, an increased handleability as compared to traditional 0D hydrogel compartments such as microcapsules. Specifically, hydrogel microcapsules are typically suspended in liquid and thus have to be handled by for instance pipetting techniques, causing microparticles to be easily lost or physically damaged due to adherence to culture plastic or pipette tips. In contrast, larger structures such as the here described 1D fibers, 2D sheets, and 3D volumes, can be handled on a macroscopic level using tools such as spatulas and/or tweezers, which allow for facile complex manipulation such as writing, weaving, or stacking of separate materials, which minimizes loss or damage.

### 2.3 Ultra‐High Throughput Production of Chondrogenic Spheroid within Cell‐Laden Compartmentalized Hydrogels

Using IAMF, large compartmentalized hydrogels can be produced at throughputs of >850‐fold higher than conventional microfluidics (10 µL min^−1^ for conventional microfluidics^[^ ^31^ ^]^ vs 8.5 mL min^−1^ for IAMF compartmentalized hydrogels). This approach could be leveraged for the mass production of cellular spheroids at an unprecedented rate (10 000 spheroids s^−1^).

**Figure 3:**
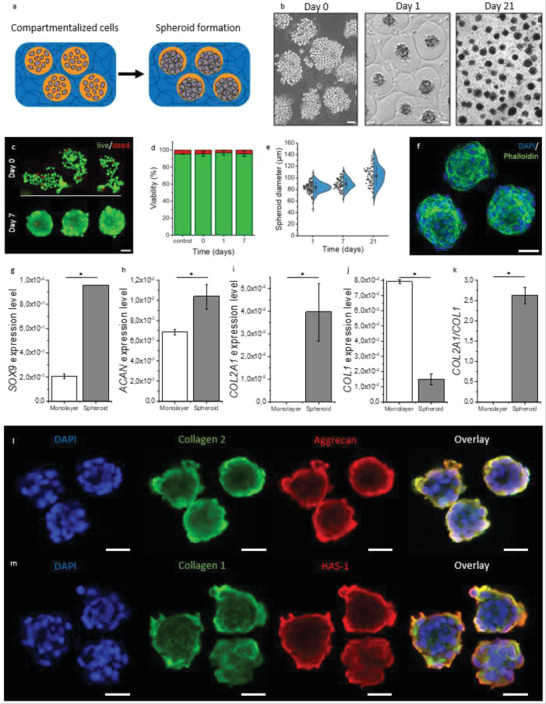
In situ spheroid formation in in‐air bioprinted compartmentalized hydrogels. a) Schematic of spheroid formation within compartmentalized hydrogel. b) Brightfield micrograph of encapsulated chondrocytes at day 0, day 1, and day 21. c) Fluorescent micrograph of viability staining at day 0 (top) and day 7 (bottom) with live cells stained green using calcein‐AM and dead cells stained red using ethidium homodimer. d) Quantification of the fraction of viable chondrocytes after 0, 1, and 7 days of culture within compartmentalized hydrogels (*n* = 136 spheroids). e) Quantification of spheroid diameter over 21 days of culture within compartmentalized hydrogels (*n* = 100). f) Confocal fluorescence micrograph of spheroids with nuclei stained blue using 4′,6‐diamidino‐2‐phenylindole (DAPI) and F‐actin stained green using Alexa fluor 488 phalloidin after 21 days of culture within compartmentalized hydrogels. g) Relative mRNA levels of the chondrogenic markers g) *SOX9*, h) *ACAN*, and i) *COL2A1*, and of the fibrocartilage marker j) *COL1*, as well as k) relative ratio between *COL2A1* and *COL1* as an indication of cartilage phenotype from human primary chondrocytes cultured as either monolayer or spheroid within compartmentalized hydrogel for 21 days (*n* = 2, 50 000 spheroids each). Fluorescence micrographs of cellular spheroids formed in compartmentalized hydrogels visualized using Immunofluorescence staining of l) DAPI (blue), collagen 2 (green), aggrecan (red), and m) DAPI (blue), collagen 1 (green), and hyaluronan synthase 1 (HAS1) (red) after 21 days of culture in chondrogenic medium. Scale bars equal 50 µm. Data are presented as mean ± SD. Significance of *p* <0.05 is indicated by *.

To demonstrate this, C20A4 chondrocytes were encapsulated in compartmentalized hydrogels by introduction of the cells in the core microjet solution (**Figure** **3**a). Upon cell‐laden compartmentalized hydrogels formation, the hydrogel was immediately washed with a surplus of culture medium and subsequently provided with fresh culture medium. Cellular spheroids formed within the hydrogel compartments within 24 h and were able to be cultured inside the compartments for at least 21 days within 3D hydrogels (Figure 3b) and 1D fibers (Figure S2, Supporting Information), which remained stable for at least 21 days of culture. Cell viability remained high upon jetting compared to the nozzle control (95.6 ± 2.8% vs 95.6 ± 0.5) (Figure 3c,d), which is to be expected since potentially harmful shear forces are largely determined by the liquid's viscosity, which in the present study is exceedingly low.^[^ ^67^ ^]^ Cell viability remained high upon subsequent cell culture (96 ± 3%) (Figure 3c,d).

The number of encapsulated cells is controlled by tuning the cell concentration in the core microjet solution, with 13 ± 5, 38 ± 10, and 73 ± 15 cells per compartment for 2 × 10^6^, 5 × 10^6^, and 10^7^ cells mL^−1^ in the core microjet solution, respectively (Figure S3, Supporting Information). Formed cellular spheroids possessed monodisperse size distributions (CV ≤ 10% at day 1), which demonstrated controlled formation of cellular spheroids within the IAMF‐produced compartmentalized hydrogels (Figure 3e,f). The ability to control the number of encapsulated cells and thus the formed spheroid diameter is especially important as spheroid diameter is known to influence biological function.^[^ ^12^ ^]^ Previous studies reported that creating 50–100 cells per spheroids improves chondrogenic performance by increasing expression of *SOX9*, *ACAN*, and *COL2A1* while lowering expression of *COL1* as compared to both single cells and larger spheroids.^[^ ^12^ ^]^ Therefore, chondrogenic experiments were performed encapsulating primary human chondrocytes at 10^7^ cells mL^−1^, which equaled 73 ± 15 cells per compartment.

Primary human chondrocytes were formed and cultured as spheroids within compartmentalized hydrogels or as monolayers in chondrogenic medium for 21 days. As compared to monolayers, chondrocytes in compartmentalized hydrogels expressed significantly higher levels of the chondrogenic master transcription factor *SOX9* (Figure 3g) as well as key cartilage extracellular matrix components *ACAN* (Figure 3h) and *COL2A1* (Figure 3i), while expressing lower levels of the undesired fibrocartilage marker *COL1* (Figure 3j) and a higher *COL2A1/COL1* ratio in the cellular spheroids as compared to monolayer cultures (Figure 3k). Combined, the gene expression profile corroborated that the cellular microspheroids that had autonomously formed within the hollow compartmentalized hydrogels indeed associated with a stronger chondrogenic phenotype than cells that were individually dispersed throughout solid hydrogels. To confirm whether true chondrogenic behavior of the spheroids in compartmentalized hydrogels had occurred, immunofluorescent staining of spheroids exposed to chondrogenic differentiation medium for 21 days was performed. Indeed, spheroids within compartmentalized hydrogels expressed cartilage markers such as collagen 1, collagen 2, aggrecan, and hyaluronan synthase 1 (Figure 3l,m). These results demonstrated that autonomously formed spheroids in compartmentalized hydrogels, which outperform conventional monolayer cultures in terms of chondrogenic performance similar to conventional low throughput produced spheroids,^[^ ^9^ ^]^ can now be considered for the engineering of cartilage, and specifically as a novel method to enable the clinical translation of the benefits of cellular spheroids in a clean, cytocompatible, single‐step, and clinically scalable manner.

### 2.4 In Air Microfluidic Spheroid‐Forming Compartmentalized Hydrogels Allows for Formation of Shape‐Stable Clinically Sized Biomaterial‐Free Cellular Tissues

**Figure 4:**
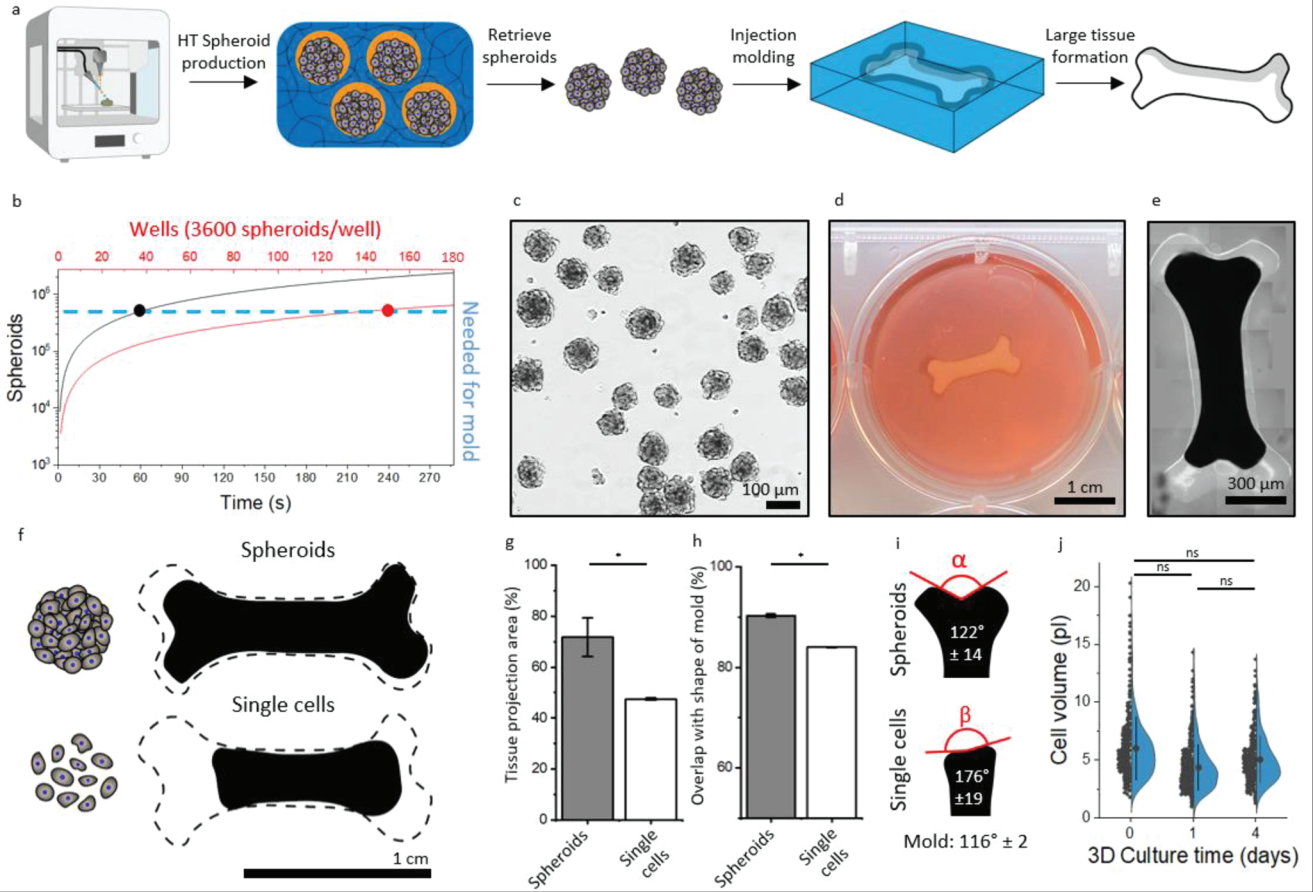
Compartmentalized hydrogel as a 3D platform for spheroid production for the engineering of clinically sized cellular tissues. a) Schematic of clinically sized cellular tissue formation, including high‐throughput (HT) spheroid production in compartmentalized hydrogel, retrieving spheroids from the compartmentalized hydrogels, injection molding of spheroids in an agarose mold, and subsequent tissue formation. b) Theoretical representation of microwells needed versus in air microfluidic runtime needed to create ±540 000 spheroids, which was required to fill the model injection mold. c) Brightfield micrograph of retrieved chondrocyte spheroids following alginate lyase treatment. d) Photograph of a centimeter‐sized tissue formed in an agarose mold in a 12‐wells plate well after 24 h of culture. e) Stitched micrograph of formed tissue after 24 h of culture. f) Projection of formed tissues produced with spheroids (top) or single cells (bottom) on top of an outline of the original mold shape. g) Tissue projection area of formed tissues produced with spheroids or single cells relative to mold projection area (*n* = 4). h) Overlap of formed tissue with spheroids or single cells relative to shape of mold (corrected for tissue projection area) (*n* = 4). i) Projection of formed tissue produced with spheroids (top) or single cells (bottom) with representation of angle in red and measured angle in white (*n* = 4). j) Cell volume of chondrocytes upon culture and spheroid formation within compartmentalized hydrogels after 0 (control), 1, and 4 days of culture (*n* = 500). Data are presented as mean ± SD. Significance of *p* <0.05 is indicated by *.

The ultra‐high throughput production of spheroid‐forming compartmentalized hydrogels allows for clinically sized engineered tissue formation. However, spheroid culture has also been found beneficial for engineering of biomaterial‐free living tissues in the form of bottom‐up tissue engineering by using spheroids as modular building blocks. In comparison with conventional single‐cell techniques, cellular spheroids allow for the production of more shape‐stable tissues.^[^ ^15^ ^]^ This tight shape and volume control is highly important in predictable tissue engineering and conformal defect filling as onset of construct shrinkage will inevitably lead to the formation of unwanted defects and cavities. Unfortunately, due to the existing spheroid batch production processes, these clinical‐sized constructs have remained out of reach due to the required amount of spheroids to form large‐scale tissue constructs; conventional spheroid production processes only offer low production rates. To address this, we hypothesized that the compartmentalized hydrogels could be sacrificed and thus act as temporary and discretionary 3D picoreactors to facilitate the mass production of monodisperse cellular spheroids. Indeed, scaling into the third dimension would significantly enhance spheroid production rate as the gold standard production platforms are all 2D in their nature. We reasoned that the ultra‐high throughput spheroid production nature of the compartmentalized hydrogel would enable the production of shape‐stable, clinical‐sized biomaterial‐free cellular tissues (**Figure** **4**a).

To produce the quantity of spheroids needed to engineer a fully cellular tissue with only a volume of 315 µL, which estimates a common full thickness cartilage defect of 1.5 cm^2^,^[^ ^68^ ^]^ would require a total of 150 individual wells of 12‐well plate (3600 spheroids per well^[^ ^9^ ^]^) (Figure 4b). The amount of trained manual labor is therefore considered nonscalable, which thus challenges wide‐spread clinical translation of spheroid‐based cellular tissues. However, utilizing the continuous IAMF compartmentalized hydrogel method, a runtime of only 60 s is needed in order to produce the same amount of spheroids (Figure 4b). It is noted that specific medical applications require a larger tissue size to be considered as clinically sized, which can easily be achieved by prolonging the production runtime from a single minute to several minutes. The evolution of multiple sequential artisan batch processes into a single continuous sub‐minute runtime process is anticipated to facilitate the feasibility of using bottom‐up engineering large tissues using cellular spheroid building blocks. Moreover, our novel approach also is more ecologically friendly as it strongly reduces the amount of plastic plates needed to create large living tissues in this bottom‐up approach.

Chondrocyte spheroids were mass produced in alginate compartmentalized hydrogels and enzymatically retrieved using an alginate lyase washing step after 4 days of culture (Figure 4c). Due to the biocompatible and orthogonal nature of alginate lyase^[^ ^69^ ^]^ and the mild washing procedure, no changes in spheroid morphology was observed. Of note, this retrieval method of cellular spheroids offers the advantage of notably milder conditions as compared to those employed in other commonly used spheroid‐forming platforms such as the forceful hydrodynamic agitation of spheroids retrieved from microwells. Approximately 540 000 spheroids or an equal total amount (4 × 10^7^) of individual cells were injected in a centimeter‐sized bone‐shaped mold to allow for the formation of a centimeter‐sized engineered tissues. The symbolic shape of a bone was chosen owing to its suitability to assess shape‐stability of formed tissues (Figure 4d,e). It was observed that utilizing spheroids for injection molding of large‐scale tissues resulted in superior shape stability as compared to single cells (Figure 4f). Specifically, mass‐produced spheroids were associated with a lower tissue shrinkage as indicated by tissue projection area, which was 71.7 ± 7.3% for spheroids and 47.5 ± 0.4% for single cells, respectively, after a single day of culture (Figure 4g). Moreover, produced tissues showed a higher overlap with the injection mold's shape when seeded with spheroids (90.3 ± 0.2%) then when single cells (84.0 ± 0.1%) were used (Figure 4h). This observation was corroborated by the measurements of the angle of the physical ends of the fully cellular bone‐shaped tissues. The curve of the bone‐shaped tissues of spheroids (122 ± 14°) maintained a high fidelity to that of the used injection mold (116 ± 2°), while the angle was virtually lost when using single‐cell suspensions (176 ± 19°) (Figure 4i). We reasoned that cellular shrinkage during the condensation phase that occurs during the formation of 3D microtissues might offer an explanation for the observed differences in the shape stability of engineered large cellular tissues. Cell volume analysis revealed that placing cells from a 2D environment into a 3D environment resulted in a cell volume decrease from 6.0 ± 2.7 to 4.4 ± 2.3 pL after a single day of culture (Figure 4j). Moreover, cell volume remained stable (5.0 ± 2.1 pL) during further 3D culture. This indicates that the initial spheroid formation in the compartmentalized hydrogels created microtissue building blocks that would—unlike individual cells from 2D cultures—not shrink when used to engineer large cellular tissues thus offering improved shape stability when used for injection mold engineering of living tissues.

## 3 Conclusion

IAMF allowed for the successful production of cell‐laden hollow compartmentalized hydrogels that enabled in situ formation of cellular spheroids in an ultra‐high throughput, clean, and single‐step manner. This innovative technology was proven to be compatible with conventional ink‐jet bioprinting technology, which facilitated the production of compartmentalized hydrogels with multiple complexities, while handheld ink‐jet bioprinting allowed for quick and easy freeform biofabrication of compartmentalized spheroid‐forming hydrogels. Utilizing the ultra‐high throughput of IAMF, the scalable production of spheroid‐based clinically sized cartilage tissues was realized in relevant timeframes, which represents a significant step toward clinical translation of cellular spheroids.

## 4 Experimental Section

### IAMF Setup

The IAMF setup for compartmentalized hydrogel was consisted of two tapered nozzles (Idex Health&Science) of similar nozzle diameter, positioned at an angle of ± 40°, which were aligned using micrometer‐precision *XYZ*‐stages (Thorlabs). The nozzles were connected to Luer‐lock glass syringes (Hamilton) with fluorinated ethylene propylene (FEP) tubing (ID 250 µm, DuPont). Flow rate was controlled by low‐pressure syringe pumps (neMESYS, Cetoni). The primary nozzle was connected to a piezoelectric actuator, which was operated at 5 Vpp with frequencies between 1 and 10 kHz, depending on the used nozzle diameter, allowing controlled break‐up of the microjet. The secondary nozzle was aligned such that its microjet coalesced with the monodisperse droplet train of the primary microjet. Controlled droplet break‐up and coalescence of the microjet with the droplet train was confirmed using a high‐speed microscope camera (SMZ800N, Nikon attached with a uEye usb camera, IDS).

### Alginate Compartmentalized Hydrogel Production and Analysis

The primary microjet was consisted of 10% w/v dextran (40 kDa, Pharmacosmos) and 50 × 10^−3^ m calcium chloride (CaCl_2_) (Sigma‐Aldrich) in dH_2_O. The secondary microjet was consisted of 0.5% w/v sodium alginate (80–120 cP, FUJIFILM Wako) and 10% v/v ethanol (EtOH) in dH_2_O. Different nozzle diameters (50, 100, 150, and 200 µm) were used to produce compartments of different compartment sizes. For 50 µm diameter nozzles, flow rates of 0.9 and 1.1 mL min^−1^ were used, respectively, for the primary and secondary microjets with an actuator frequency of 6.5 kHz. For 100 µm diameter nozzles, flow rates of 2 and 2.2 mL min^−1^ were used, respectively, for the primary and secondary microjets with an actuator frequency of 5.5 kHz or flow rates of 4 and 4.5 mL min^−1^ were used, respectively, for the primary and secondary microjets if indicated that the total flow rate was 8.5 mL min^−1^ with an actuator frequency of 5.5 kHz. For 150 µm diameter nozzles, flow rates of 3 and 3.2 mL min^−1^ were used, respectively, for the primary and secondary microjets with an actuator frequency of 4.5 kHz. For 200 µm diameter nozzles, flow rates of 4 and 4.2 mL min^−1^ were used, respectively, for the primary and secondary microjets with an actuator frequency of 3.5 kHz. The microjets were characterized by We ± 25 using collisional angles of ± 40°, which corresponded to We_impact_ ± 10.

Compartmentalized hydrogels were produced using multiple fabrication options. For the first option, nozzles were fixed in position and the in‐air‐formed compartments were collected in a petri dish on a moveable *XZ* collection stage for the production of compartmentalized hydrogels. For the second option, nozzles were fixed upon the moveable printhead (1 m s^−1^) of a 3D printer (Inkredible+, CELLINK) and the in‐air‐formed compartments were collected in a petri dish for the production of compartmentalized hydrogels. For the third option, nozzles were fixed upon a handheld device, allowing manual deposition of in‐air‐formed compartments for the production of compartmentalized hydrogels. Regardless of which setup was being used, 1D fibers, 2D planes, and 3D volumes of compartmentalized hydrogel could be produced by controlled movement of either nozzles or collection petri dish.

Compartmentalized hydrogels were visualized using brightfield microscopy (EVOS FL Imaging System, ThermoFisher). Compartment diameter was measured using the Feret diameter. The hollow nature of the compartments was investigated via the addition of 0.5 mg mL^−1^ 2000 kDa Dextran‐TRITC (Sigma‐Aldrich) to the primary microjet and 0.5 mg mL^−1^ 2000 kDa Dextran‐FITC (Sigma‐Aldrich) to the alginate containing secondary microjet. Fluorescently labeled compartmentalized hydrogels were analyzed using confocal *z*‐stack microscopy analysis (Nikon A1 confocal). Size distribution, monodispersity, angle orientation, ellipsoid elongation, and fluorescent intensity were analyzed using ImageJ software.

### Cell Culture

C20A4 chondrocytes (human chondrocyte cell line, Sigma Aldrich, SCC041) were cultured in Dulbecco's modified Eagle medium (DMEM, Gibco) with 10% fetal bovine serum (FBS, Sigma‐Aldrich), 100 U mL^−1^ penicillin (Gibco), and 100 µg mL^−1^ streptomycin (Gibco). Culture medium was changed biweekly. Human chondrocytes were cultured in proliferation medium containing DMEM, 10% FBS, 100 U mL^−1^ penicillin, 100 µg mL^−1^ streptomycin, 0.1 × 10^−3^ m L‐proline (Sigma‐Aldrich), 1% nonessential amino acids (NEAA, Sigma‐Aldrich), and 1% ascorbic acid (ASAP, Sigma‐Aldrich), or in chondrogenic medium containing DMEM, 100 U mL^−1^ penicillin, 100 µg mL^−1^ streptomycin, 0.1 × 10^−3^ m L‐proline (Sigma‐Aldrich), 1% nonessential amino acids (NEAA, Sigma‐Aldrich), and 1% ascorbic acid (ASAP, Sigma‐Aldrich), 10 ng mL^−1^ TGFb3 (R&D systems), and 0.1 × 10^−6^ m dexamethasone (Sigma‐Aldrich). Cells were passaged when 80% confluency was reached. Cell cultures were kept in a humidified environment at 37 °C with 5% CO_2_.

### Cell Encapsulation

When preparing cell‐laden compartmentalized hydrogels, dH_2_O in the microjet solutions was replaced with DMEM without phosphates (Gibco). Cells were detached using trypsin‐ethylenediaminetetraacetic acid (Invitrogen), washed with medium and subsequently flown through a 40 µm cell strainer (EASYstrainer, Greiner) to ensure a single‐cell suspension. Cells were then suspended at 10^7^ cells mL^−1^ in the primary microjet solution unless stated otherwise. The cell‐laden solution was then loaded into and kept in an ice‐cooled gastight syringe for the duration of the encapsulation procedure (<10 min). Nozzle diameters of 100 µm were used in cell encapsulation experiments, with flow rates of 2 and 2.2 mL min^−1^ for the primary and secondary microjets, respectively, with a frequency of 5.5 kHz applied by the piezo‐actuator. Cell‐laden compartmentalized hydrogels were collected in a petri dish or directly into culture plates. Immediately after production, the compartmentalized hydrogel was washed with medium in order to wash away excess ethanol to maintain high cell viability. Finally, fresh culture medium was added upon which the compartmentalized hydrogels were placed in culture.

Cellular aggregation upon cell encapsulation was monitored by brightfield microscopy (EVOS FL Imaging System, ThermoFisher). Cell viability was studied by staining with calcein AM and ethidium homodimer‐1 according to manufacturer's protocol (Invitrogen) and imaging using a digital fluorescence microscope (EVOS FL Imaging System, ThermoFisher).

For additional analyses, cell‐laden compartmentalized hydrogels were washed with phosphate‐buffered saline (PBS) and fixated using a 10% buffered formalin solution (Sigma‐Aldrich). Cells were permeabilized using 0.1% Triton X‐100 (Sigma‐Aldrich) and were subsequently stained with 2.5 U mL^−1^ phalloidin‐AF488 (Thermo Fisher Scientific) and 10 µg mL^−1^ DAPI (Thermo Fisher Scientific) to stain F‐actin and nuclei, respectively. Fluorescently stained samples were analyzed using confocal microscopy (Nikon confocal A1) and ImageJ software.

### Gene Expression Analysis

Primary human chondrocytes were encapsulated in compartmentalized hydrogels and cultured for 21 days in chondrogenic medium. As a control, primary human chondrocytes were cultured in monolayer. Samples for quantitative polymerase chain reaction (qPCR) analysis were prepared with TRIzol (Invitrogen) lysis buffer and processed for RNA isolation using the miRNeasy kit (QIAGEN) following the protocol provided by the supplier. cDNA was synthesized using the iScript cDNA synthesis kit (Bio‐Rad). cDNA was then subjected to qPCR using SensiMix SYBR & fluorescein kit (Bioline) on a CFX Connect Real‐time System (Bio‐Rad).

### Cartilage Production Analysis

Primary human chondrocytes were encapsulated in compartmentalized hydrogels and cultured for 21 days in chondrogenic medium. Spheroids were fixated using 10% buffered formalin solution, permeabilized using 0.1% Triton‐X (Sigma‐Aldrich), blocked using 10% bovine serum albumin (Sigma‐Aldrich), and stained using 1:100 anti‐collagen 1 (Novus biological), 1:100 anti‐collagen 2 (Abcam), 1:100 anti‐aggrecan (Abcam), or 1:200 anti‐hyaluronansynthase 1 (Abcam), in combination with 1:250 AF488 (Invitrogen) and 1:200 AF647 (Abcam) labeled secondary antibodies, and 1:100 DAPI as counter staining.

### Large Cellular Tissue Formation

C20A4 cells were encapsulated in compartmentalized hydrogels and cultured for four days, upon which the cellular spheroids were retrieved by PBS washing and 10 U mL^−1^ alginate lyase (Sigma‐Aldrich) incubation for 30 min at 37 °C. Approximately 540 000 spheroids (±4 × 10^7^ cells) were injected into an agarose bone‐shaped mold to form a large cellular tissue. As a control, 4 × 10^7^ single cells were injected in the bone mold. The total mold volume in which the cells could be injected was 315 µL, and was produced as previously described.^[^ ^70^ ^]^ Agarose molds were incubated in culture medium for 24 h prior to use. Formed large tissues were analyzed using macroscopic photography (Canon EOS 6D) and brightfield microscopy (EVOS FL Imaging System, ThermoFisher). Cell size/volume was analyzed by brightfield imaging upon trypsinization of spheroids that were cultured for 0, 1, or 4 days. Large tissue surface area, shape, and cell volume were analyzed using ImageJ software.

### Statistical Analysis

Data were presented as mean ± SD. Sample size per experiment was reported in figure descriptions. Significance was determined based on one‐way analysis of variance analysis. Significance of *p* <0.05 was indicated by *. All statistical analyses were performed in OriginPro2017.

## Conflict of Interest

The authors declare no conflict of interest.

## Author Contributions

Conception: B.v.L. and J.L. Experimental design: B.v.L., M.S., M.G., T.K., and J.L. Data interpretation: B.v.L. and J.L. Manuscript was written by B.v.L. and J.L.

## Supporting information

Supporting Information

Supplemental Movie 1

## Data Availability

The data that support the findings of this study are available from the corresponding author upon reasonable request.
