# Supplementary material for: Single‐Step Biofabrication of In Situ Spheroid‐Forming Compartmentalized Hydrogel for Clinical‐Sized Cartilage Tissue Formation
Source: Adv Healthc Mater. 2023 Oct 26;13(2):2300095. doi: 10.1002/adhm.202300095 (PMC11468307; doi:10.1002/adhm.202300095)
Supplement: Supplementary file 1 — Supporting Information [file ADHM-13-2300095-s002.pdf]

ADVANCED HEALTHCARE MATERIALS

Supporting Information

for *Adv. Healthcare Mater.*, DOI 10.1002/adhm.202300095

Single-Step Biofabrication of In Situ Spheroid-Forming Compartmentalized Hydrogel for Clinical-Sized Cartilage Tissue Formation

*Bas van Loo, Maik Schot, Melvin Gurian, Tom Kamperman and Jeroen Leijten**

Supplemental:

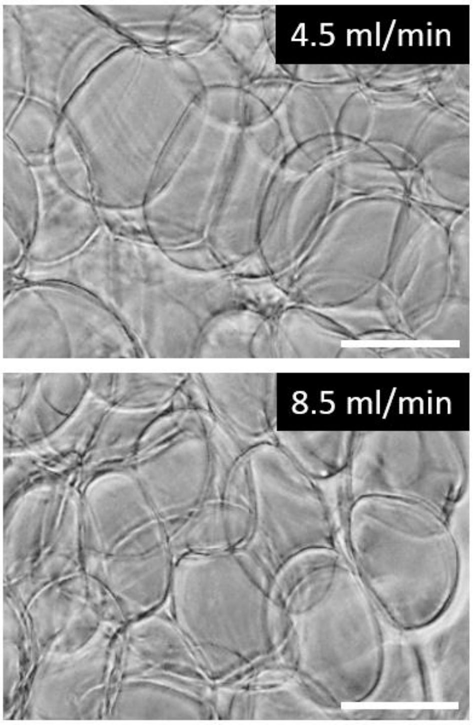

Figure S1. Compartmentalized hydrogel produced at different total flowrates. Brightfield micrographs of compartmentalized hydrogels produced with a total flow rate of 4.5 ml/min and 8.5 ml/min. Scale bars equal 300 µm.

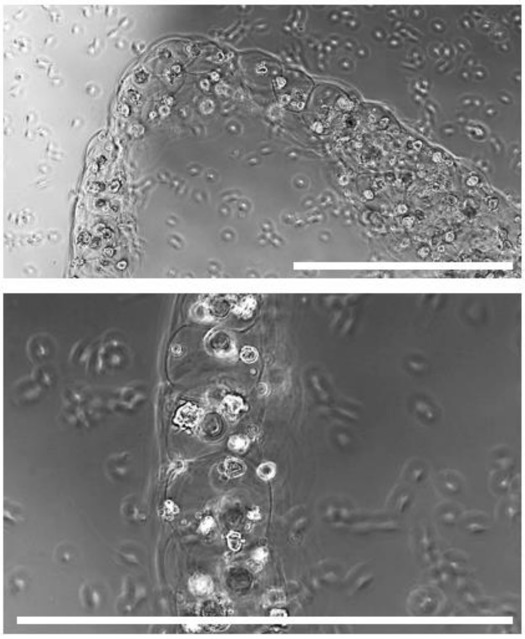

Figure S2. Spheroid formation in compartmentalized fibers. Brightfield micrographs of spheroid formation in compartmentalized fibers. Scale bars equal 1000 µm.

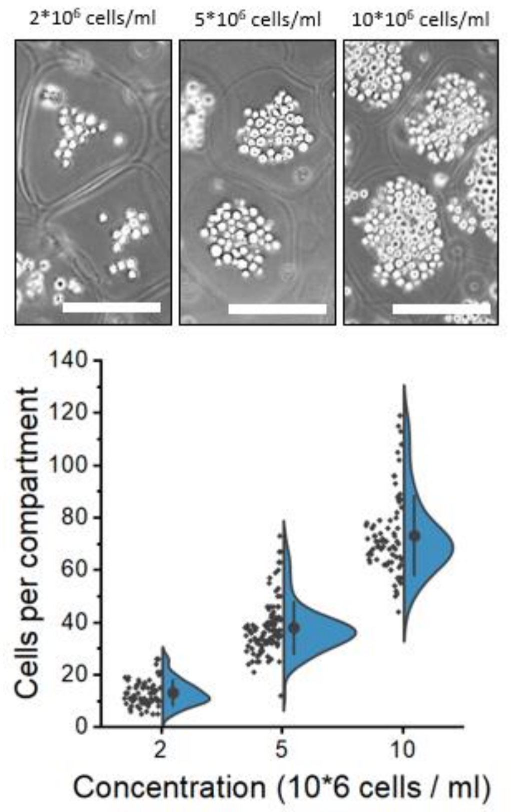

Figure S3. Control over amount of encapsulated cells by tuning cell concentration. (a) Brightfield micrographs of encapsulated cells at $2*10^6$, $5*10^6$, and $10*10^6$ cells/ml. (b) Quantification of cells per compartment (n=72). Scale bars equal 200 µm. Data is presented as mean ± SD.

Movie S1. In Air microfluidic ink-jet printing of tube-like compartmentalized hydrogel.
